# Comparison of the expression and function of Lin28A and Lin28B in colon cancer

**DOI:** 10.18632/oncotarget.12869

**Published:** 2016-10-25

**Authors:** Tianzhen Wang, Yan He, Yuanyuan Zhu, Mingwei Chen, Mingjiao Weng, Chao Yang, Yan Zhang, Ning Ning, Ran Zhao, Weiwei Yang, Yinji Jin, Jing Li, Riju James Rajkumar Ezakiel Redpath, Lei Zhang, Xiaoming Jin, Zhaohua Zhong, Fengmin Zhang, Yunwei Wei, Guomin Shen, Dong Wang, Ying Liu, Guangyu Wang, Xiaobo Li

**Affiliations:** ^1^ Department of Pathology, Harbin Medical University, Harbin, China; ^2^ Department of Gastrointestinal Medical Oncology, the Affiliated Tumor Hospital of Harbin Medical University, Harbin, China; ^3^ Department of Anatomy, Harbin Medical University, Harbin, China; ^4^ Department of Nutrition and Food Hygiene, Public Health College, Harbin Medical University, Harbin, China; ^5^ Department of Gastrointestinal Surgery, International Hospital of Pecking University, Beijing, China; ^6^ Department of Microbiology, Harbin Medical University, Harbin, China; ^7^ Department of General Surgery, the First Affiliated Hospital of Harbin Medical University, Harbin, China; ^8^ Department of Medical Genetics, Medical College, Henan University of Science and Technology, Luoyang, China; ^9^ College of Bioinformatics Science and Technology, Harbin Medical University, Harbin, China; ^10^ The Northern Medicine Translational Center, Heilongjiang Province Academy of Medical Science, Harbin, China

**Keywords:** colon cancer, Lin28A, Lin28B

## Abstract

Lin28A and Lin28B are highly conserved RNA binding proteins with similar structure and functions. Recent studies demonstrated that both of them act as oncogenes and promote cancer progression. However, few researches compared the expression and functions of both oncogenes in human malignant tumors at same time. Additionally, although the expression and role of Lin28B in colon cancer is frequently reported, the expression and functions of Lin28A in colon cancer are largely unknown. In this study, we have systematically evaluated the expressional pattern, mutation status and correlation of both Lin28A and Lin28B in colon cancer tissues for the first time, and compared the roles of Lin28A and Lin28B in the proliferation, migration, invasion and apoptosis of colon cancer cells in vitro. We have showed that they are co-expressed and have functional similarities, however, the molecular mechanisms underlying their similar functions may not be identical. This study contributes to clarify the similarities and differences of Lin28A and Lin28B in colon cancer progression.

## INTRODUCTION

Lin28A and Lin28B are highly conserved RNA binding proteins with similar structure and functions. Lin28A was first discovered in nematode Caenorhabditis elegans, and was proved to regulate the developmental timing [1, 2], whereas Lin28B was first discovered in hepatocellular carcinoma(HCC), where it is highly expressed [3]. Numerous studies have shown that both Lin28A and Lin28B are over-expressed in many cancer types [4, 5]. Further researches have shown that both Lin28A and Lin28B function as oncogenes in a variety of human cancers either by suppressing the biogenesis of microRNA let-7s or by stabilizing the oncogenic transcripts [6], the upregulation of either Lin28A or Lin28B proteins was associated with malignant biological behaviors and poor prognosis in cancer patients [6].

Although Lin28A and Lin28B share similarities in many characters and functions in cancer development and progression, such as suppressing the biogenesis of let-7 family miRNAs, promoting the proliferation and metastasis of malignant cancer cells [6], there are still some differences about their expression and functions. For example, the subcellular distribution of Lin28A and Lin28B is different. Lin28A is predominantly localized in the cytoplasm, whereas Lin28B exclusively resides in the nucleus [7]. The mechanism of inhibiting the biogenesis of let-7 is also different for Lin28A and Lin28B. Lin28A blocks the pre-let-7 processing by Dicer via recruiting TUT4, whereas Lin28B interacts with pri-let-7 and inhibits its processing by the Microprocessor [7]. Additionally, RNA binding protein Lin28A and Lin28B interacts with different target mRNAs in cancer cells [6]. These researches imply the importance to distinguish the expression and functions of both Lin28A and Lin28B in certain cancer types.

Although it was demonstrated that either Lin28A or Lin28B is over-expressed in malignant tumors and promotes cancer progression, few researches detected the expression and function of both oncogenes in human malignant tumors at same time. For instance, several studies showed that Lin28B is overexpressed in colon cancer and promotes colon cancer progression [8, 9], whereas the expression and functions of Lin28A in colon cancer are largely unknown.

In this study, we have systematically evaluated the expressional pattern and correlation of both Lin28A and Lin28B in colon cancer tissues, and have determined and compared the roles of Lin28A and Lin28B in the proliferation, migration, invasion and apoptosis of colon cancer cells in vitro. This study contributes to clarify the similarities and differences of Lin28A and Lin28B in colon cancer progression.

## RESULTS

### The expression pattern of Lin28A and Lin28B in colon cancer

**Figure 1:**
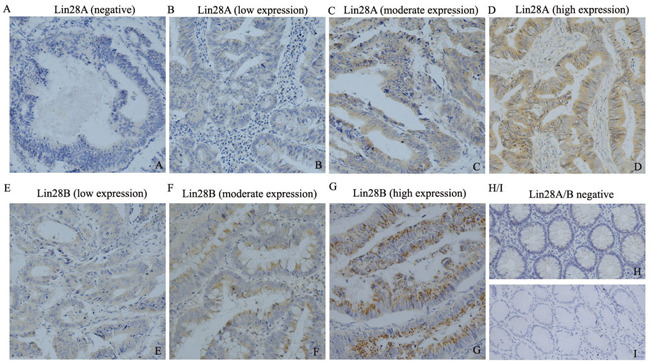
The expression pattern of Lin28A and Lin28B in colon cancer tissues detected by immunohistochemistry (200×) **A-D.** illustrates the expression of Lin28A in colon cancer tissues variated from negative to high expression; whereas **E-G.** illustrates the expression of Lin28B in colon cancer tissues variated from low to high expression (n=65). **H** and **I.** illustrate the negative expression of Lin28A and Lin28B in normal colon tissues (n=10) respectively.

**Figure 2:**
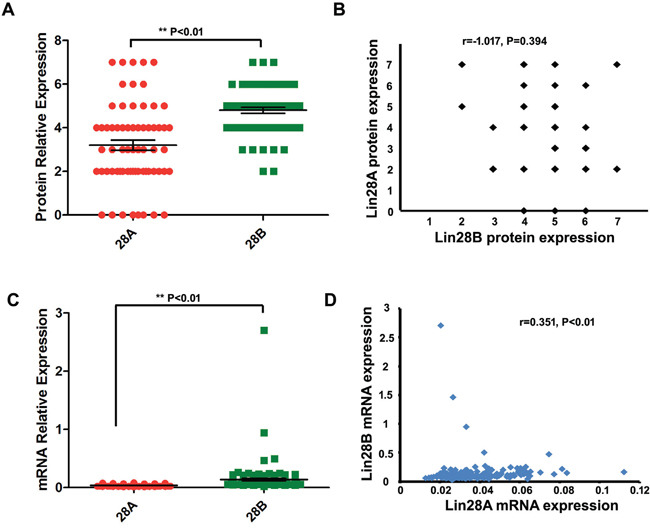
Comparison and correlational analysis of the expression of Lin28A and Lin28B in colon cancer samples **A.** The protein expression of Lin28B is significantly higher than that of Lin28A in colon cancer tissues (P<0.01) (n=65). **B.** The protein level of Lin28A is not correlated with that of Lin28B in the same colon cancer tissues (P>0.05) (n=65). **C.** The mRNA expression of Lin28B is significantly higher than that of Lin28A in colon cancer tissues (P<0.01) (n=162). **D.** The mRNA level of Lin28A is significantly correlated with that of Lin28B in the same colon cancer tissues (r=0.351, P<0.01) (n=162).

To learn the expression pattern of Lin28A and Lin28B in colon cancer, we initially detected the expression of both oncogenic proteins in 65 colon cancer tissues and 10 normal tissues using immunohistochemistry staining. The results showed that Lin28B protein is expressed in all the observed colon cancer tissues samples (positive rate is 100%), whereas Lin28A protein is expressed in 57 out of 65 samples (positive rate is 87.7%) (Figure 1). Not just the expressional frequency of Lin28B protein is higher than that of Lin28A (p<0.01) but the expressional level of Lin28B protein is also significantly higher than the protein level of Lin28A (Figure 2A, P<0.01). However, both Lin28A and Lin28B were negative in normal tissues. The mRNA expression of both Lin28A and Lin28B was also analyzed in 162 colon cancer patients, and the results showed that the mRNA level of Lin28B is also significantly higher than mRNA level of Lin28A in colon cancer (Figure 2C, P<0.01). These results suggest that both Lin28A and Lin28B are expressed in colon cancer, but the expression level of Lin28B is higher than that of Lin28A.

**Figure 3:**
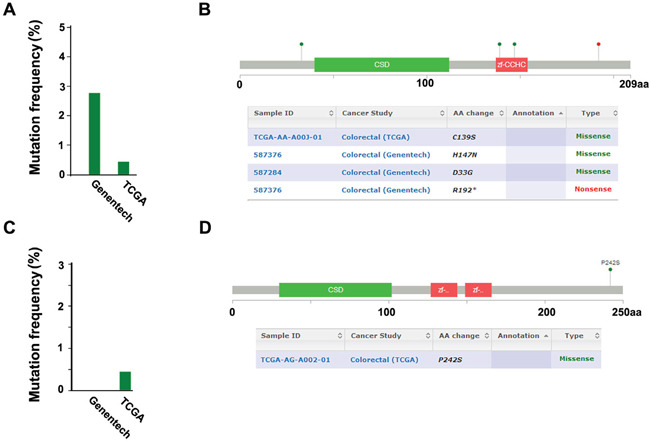
Detection of the mutation of Lin28A and Lin28B in colon cancer samples based on the cBioPortal cancer genomic data **A.** The mutation frequency of Lin28A (n=703, 631 from TCGA and 72 from Genentech). **B.** The mutation types and sites of Lin28A protein. **C.** The mutation frequency of Lin28B (n=703, 631 from TCGA and 72 from Genentech). **D.** The mutation types and sites of Lin28B protein.

Furthermore, to investigate the relationship between the expressional level of both Lin28A and Lin28B in the same patient, we have analyzed the correlation between Lin28A and Lin28B in all of the patient's samples, and the result revealed that there is no significant correlation between the protein level of Lin28A and Lin28B in the same colon cancer sample (Figure 2B, r=−0.107, P>0.05), however, the mRNA level of Lin28A and Lin28B showed a significant positive correlation (Figure 2D, r=0.351, P<0.01). Additionally, we have also evaluated the mutation status of Lin28A and Lin28B in colon cancer. Based on the large-scale cancer genomics data sets provided by cBioPortal (http://www.cbioportal.org/), we analyzed two colorectal adenocarcinoma data sets, one set containing 631 samples from TCGA, and another set containing 72 samples from Genentech, and have found that both Lin28A and Lin28B have mutations in colon cancer samples, however, the mutation frequency is different, the mutation of Lin28A is detected from both Genentech and TCGA data sets with a frequency of 2.3% and 0.4% respectively (Figure 3A); whereas the mutation of Lin28B is only detected from TCGA data set with a frequency of 0.4% (Figure 3C). The mutation types of Lin28A are nonsense mutation (R192*) and missense mutations in multi-sites (D33G, C139S and H147N) (Figure 3B); whereas Lin28B only has one missense mutation (P242S) (Figure 3D).

### Both Lin28A and Lin28B are predominantly distributed in cytoplasm of colon cancer cells

**Figure 4:**
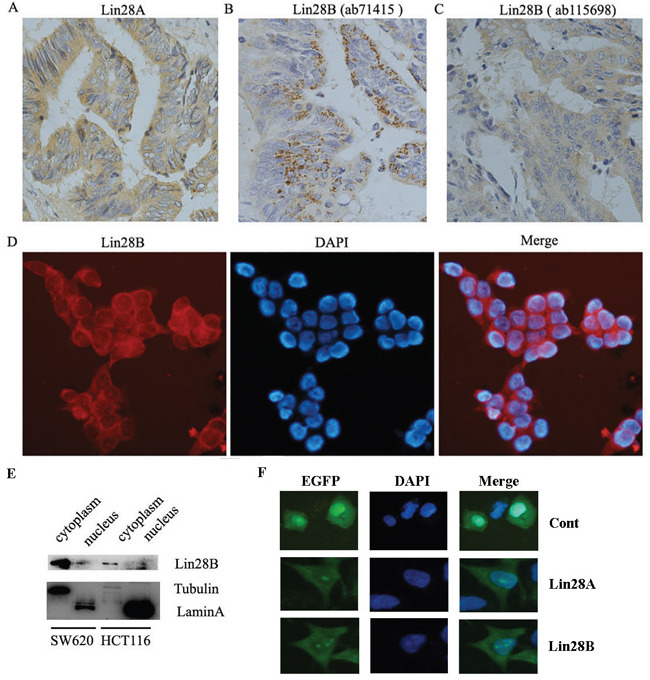
Lin28A and Lin28B are predominantly distributed in the cytoplasm of colon cancer cells **A.** Lin28A was distributed in the cytoplasm of colon cancer cells (×400). **B** and **C.** The cytoplasm distribution of Lin28B in colon cancer tissues illustrated by immunohistochemistry with different antibody against-Lin28B (×400). **D.** The cytoplasm distribution of Lin28B in SW620 cells illustrated by immunofluorescence (×400). **E.** The cytoplasm distribution of Lin28B in SW620 and HCT116 cells illustrated by subcellular proteins immunoblot. **F.** The cytoplasm distribution of Lin28A-EGFP and Lin28B-EGFP in HCT116 cells detected by fluorescent microscope (×400).

It has been reported that the cellular distribution of Lin28A protein and Lin28B protein is different, Lin28A protein is predominantly localized in the cytoplasm whereas Lin28B protein exclusively expressed in the nuclei [7]. In this study, we have found that the expression of Lin28A in colon cancer tissues is localized in the cytoplasm (Figure 4A), which is consistent with previous study. However, the expression of Lin28B in all of the observed colon cancer tissues is also predominantly localized in the cytoplasm instead of the nuclei (Figure 4B & 4C), which is different from the previous observation. To exclude the possibility of false positive staining resulting from the non-specific reaction of antibody with other antigens, we used an additional Lin28B antibody to detect the expression of Lin28B using immunohistochemistry. And the results from different antibody (ab71415 & ab115698) showed the same subcellular distribution of Lin28B protein (Figure 4B & 4C). Further, we detected the subcellular distribution of Lin28B protein in colon cancer cell lines. The immunofluorescent assay showed that Lin28B is predominantly located in the cytoplasm of SW620 cells (Figure 4D). The subcellular fractionation assay also demonstrated that Lin28B majorly distributed in the cytoplasm of both HCT116 and SW620 cells (Figure 4E). Additionally, we exogenously expressed Lin28B-EGFP fusion protein in HCT116 cells to track the distribution of Lin28B. The result showed that Lin28B-EGFP fusion protein is also predominately located in the cytoplasm of the cells, while EGFP is distributed throughout the cells (Figure 4F). Analyzing together, we demonstrated that Lin28B is predominantly located in the cytoplasm of colon cancer cells, which is contradictory to the previous report.

### The impact of Lin28A and Lin28B on clinical characteristics

**Figure 5:**
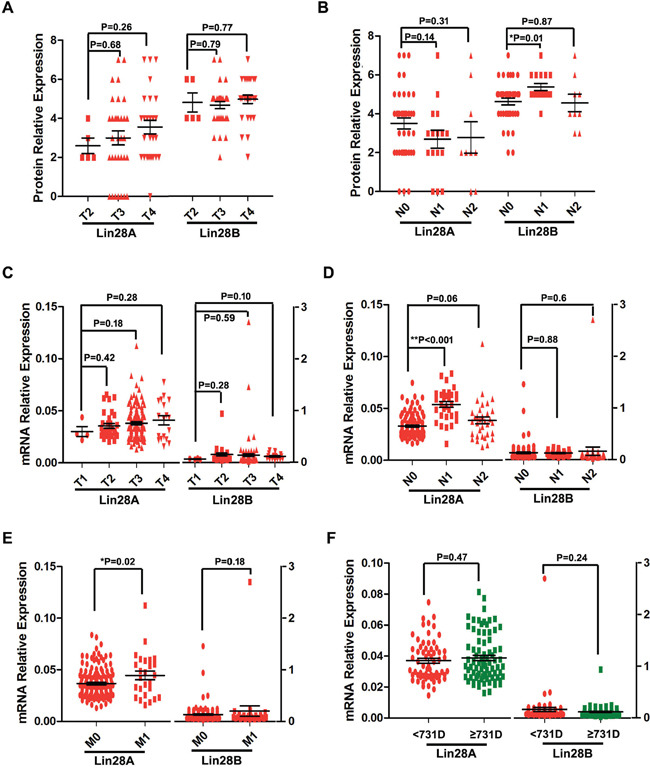
Analysis of the impact of the expression of Lin28A or Lin28B on the tumor stages and prognosis of colon cancer patients **A.** Neither Lin28A nor Lin28B protein expression is significantly different among different tumor size stages. **B.** The protein level of Lin28B but not Lin28A is significantly higher in patients with lymph node metastasis (N1 but not N2) than patients with non-lymph node metastasis (N0) (P<0.05). **C.** Neither Lin28A nor Lin28B mRNA level is significantly different among different tumor size stages. **D.** The mRNA level of Lin28A but not Lin28B is significantly higher in patients with lymph node metastasis (N1 but not N2) than patients with non-lymph node metastasis (N0) (P<0.01). **E.** The mRNA level of Lin28A instead of Lin28B in colon cancer patients with remote organ metastasis is significantly higher than that of patients with non-metastasis (P<0.05). **F.** The mRNA level of either Lin28A or Lin28B is not associated with the prognosis of colon cancer patients.

Next, we investigated the impact of Lin28A and Lin28B on the clinical characters of colon cancer, by comparing the expression of Lin28A or Lin28B in different stages of tumor size or metastasis of colon cancer. Our results showed that Lin28A protein level is elevated with increase in tumor size, even though the difference between them is not significant, whereas the expression of Lin28B protein has not shown any difference between different tumor size stages (Figure 5A). The mRNA level of either Lin28A or Lin28B also has no difference for different tumor size stages (Figure 5C). These results suggested that neither Lin28A nor Lin28B correlates with tumor size stages. So, The result about the expression of either Lin28A or Lin28B in different lymph node metastasis stages showed that Lin28B protein level is significantly higher in patients with lymph node metastasis (N1 but not N2) than patients with non-lymph node metastasis (N0) (Figure 5B), whereas the Lin28A mRNA is significantly increased in patients with lymph node metastasis (N1 but not N2) than patients with non-lymph node metastasis (N0) (Figure 5D). Additionally, the mRNA level of Lin28A instead of Lin28B in colon cancer patients with remote organ metastasis is significantly higher than that of patients with non-metastasis (Figure 5E). These results imply that both Lin28A and Lin28B are involved in the colon cancer metastasis. Furthermore, we detected the impact of the expression of either Lin28A or Lin28B on the prognosis of colon cancer patients. According to the median of survival time (731 days) of all patients, we divided these patients as two groups, one was termed as long survive group with survival time equal or more than 731 days, while another was defined as short survive group with survival time less than 731 days. And the result showed that the expression of neither Lin28A nor Lin28B is significantly associated with the prognosis of colon cancer patients (Figure 5F).

### Both Lin28A and Lin28B promote the migration and invasion of colon cancer cells

**Figure 6:**
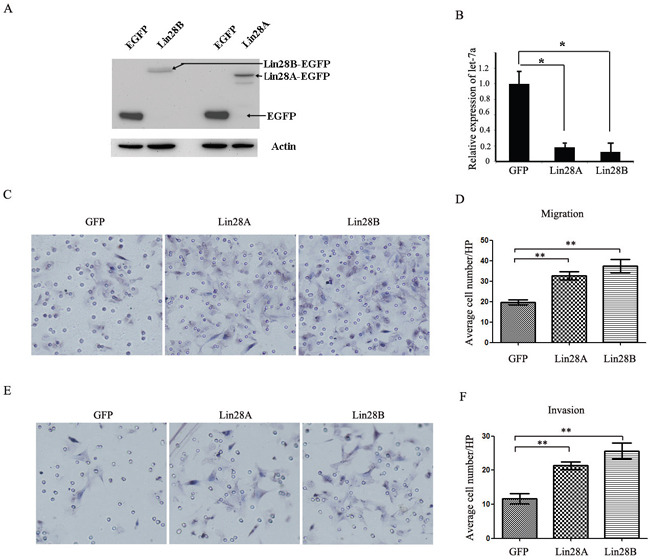
Both Lin28A and Lin28B promote the migration and invasion of HCT116 cells **A.** The overexpression of exogenic Lin28A-GFP and Lin28B-GFP fusion protein in HCT116 cells detected by western blot. **B.** The over-expression of either Lin28A or Lin28B inhibits the generation of mature let-7a in HCT116 cells (*: P<0.05). **C** and **D.** Both Lin28A and Lin28B promote the migration of HCT116 cells (**: P<0.01). **E** and **F.** Both of Lin28A and Lin28B promote the invasion of HCT116 cells (**: P<0.01).

Since the expression of both Lin28A and Lin28B is associated with colon cancer metastasis, we evaluated and compared the influence of both onco-proteins on the migration and invasion of HCT116 cells in vitro. Initially we employed lentiviral vectors to over-express Lin28A and Lin28B in HCT116 cells respectively, and the exogenous expression of Lin28A-GFP and Lin28B-GFP has been detected by Western blot (Figure 6A), whereas detecting the expression of let-7a confirmed that both Lin28A-GFP and Lin28B-GFP fusion proteins are functional (Figure 6B) and the trans-well experiments showed that both Lin28A and Lin28B enforced expression significantly promotes the migration (Figure 6C & 6D) and invasion of HCT116 cells (Figure 6E & 6F).

### Both Lin28A and Lin28B promote the proliferation of colon cancer cells

**Figure 7:**
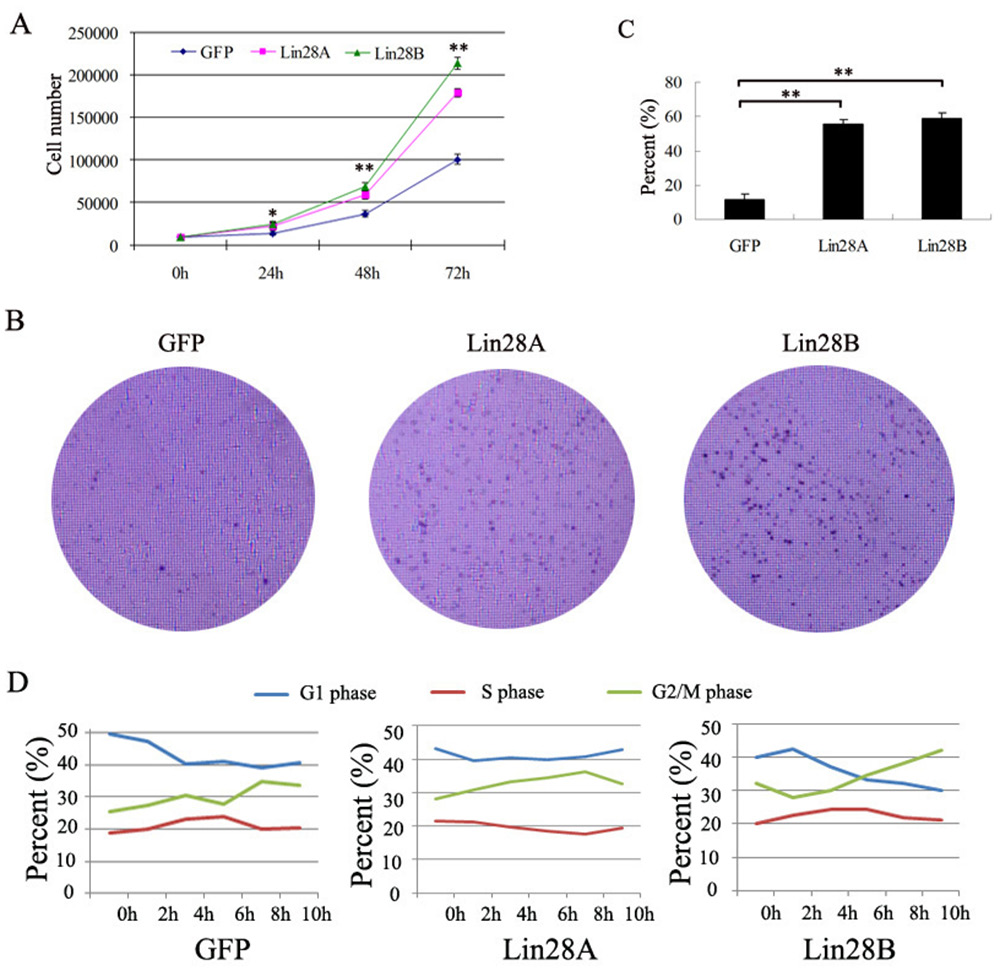
Both Lin28A and Lin28B promote the proliferation of HCT116 cells **A.** Both of Lin28A and Lin28B promote the proliferation of HCT116 cells detected by cell growth curve assay (*: P<0.05; **: P<0.01). **B** and **C.** Both of Lin28A and Lin28B promotes the proliferation of HCT116 cells detected by clone formation assay (**: P<0.01). **D.** The cell cycle assay demonstrated that Lin28A promotes the transition of cell cycle from S phase to G2/M phase, whereas Lin28B facilitates the transition of cell cycle from both G1 phase to S phase and S phase to G2/M phase.

Even though the clinical character's analysis did not show the correlation between both onco-proteins and tumor size stages, numerous experiments suggested that over-expression of Lin28 in malignant tumors promotes the proliferation of cancer cells. Then we evaluated the effect of Lin28A and Lin28B on the proliferation of colon cancer cells by performing the cell growth curve assay and colony formation assay. By counting the cells at different time points, we showed that enforced expression of either Lin28A or Lin28B has significantly increased the cell number of HCT116 cells (Figure 7A). The clone formation assay has also demonstrated that over-expression of either Lin28A or Lin28B has significantly enhanced the clone formation of HCT116 cells (Figure 7B & 7C). The cell cycle analysis showed that over-expression of Lin28A promotes the transition of cell cycle from S phase to G2/M phase, whereas over-expression of Lin28B facilitates the transition of cell cycle from both G1 phase to S phase and S phase toG2/M phase (Figure 7D). These results suggested that either Lin28A or Lin28B could promote the proliferation of colon cancer cells but mechanisms may vary.

### Both Lin28A and Lin28B enhance the apoptosis of colon cancer cells induced by 5-Fu

**Figure 8:**
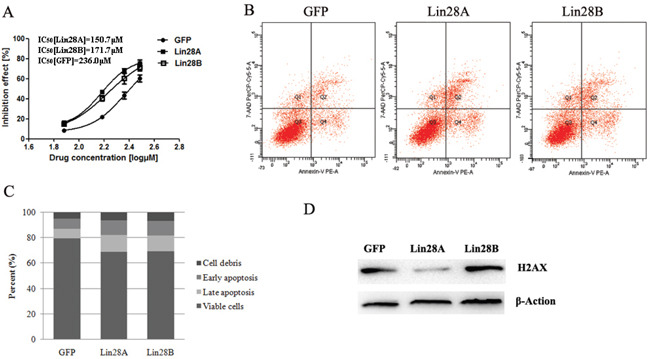
Both Lin28A and Lin28B enhance the apoptosis of HCT116 cells induced by 5-Fu **A.** Both Lin28A and Lin28B promote the chemosensitivity of HCT116 cells to 5-Fu. **B** and **C.** Both of Lin28A and Lin28B enhance the apoptosis of HCT116 cells induced by 5-Fu. **D.** Lin28A instead of Lin28B suppresses the expression of H2AX.

We previously found that Lin28A elevated expression enhanced the chemosensitivity of colon cancer cells to 5-Fu by promoting cell apoptosis in a let-7 independent manner [6]. Then we compared the effect of both onco-proteins on the chemosensitivity of colon cancer cells to 5-Fu in this study. The results showed that the over-expression of both Lin28A and Lin28B could enhance the chemotherapy sensitivity of HCT116 cells to 5-Fu (Figure 8A), and further studies showed that both onco-proteins enhanced the apoptosis of HCT116 cells induced by 5-Fu (Figure 8B & 8C). We previously demonstrated that suppressing the expression of DNA damage repairing gene H2AX contributed to the effect of Lin28A facilitating apoptosis of colon cancer cells induced by 5-Fu, then we observed the influence of both onco-proteins on the expression of H2AX in HCT116 cells, and the result revealed that Lin28A but not Lin28B inhibiting the expression of H2AX (Figure 8D), which implies that Lin28A and Lin28B enhances the chemosensitivity of colon cancer cells to 5-Fu via different mechanisms.

## DISCUSSION

In this study, we have evaluated the co-expression of Lin28A and Lin28B in colon cancer tissues for the first time, and we showed that both oncogenes are expressed in colon cancer tissues, and the expression of Lin28B was significantly higher than the expression of Lin28A. Further analysis showed a negative correlation between both onco-proteins, this result is similar as previous report in which Lin28A and Lin28B is reversely expressed in breast cancer [7]. We also analyzed the correlation of mRNA expression between both oncogenes and showed a significant positive correlation between them. These results suggested an inconsistence between the mRNA and protein level of both oncogenes, the inconsistence may have resulted from the variation of different patient groups or from the post-transcriptional regulation for their expression, however, it needs further experimentation to be clarified. Additionally, for the first time, we have reported the mutation status of both oncogenes. According to our results, it seems that the mutation frequency of Lin28A is more frequently than that of Lin28B in colon cancer.

Up to date, it is still not clear about the subcellular localization of Lin28B. Originally, Lin28B was found predominantly in the cytoplasm in Huh7 cells [3]. Two more studies also indicated the cytoplasmic expression of Lin28B using immunohistochemistry [8, 9]. However, one recent research showed that Lin28B is exclusively located in the nucleus [7], and some other studies demonstrated that Lin28B predominantly distributes in the nucleus of certain cancer cells [7, 10–12]. In this study, we have confidently demonstrated that Lin28B is majorly expressed in the cytoplasm instead of the nucleus in colon cancer cells.

In 2011, Catrina et al. first reported the overexpression of Lin28B in colon cancer, and showed that overexpression of Lin28B can promote colon cancer progression and be correlated with poor prognosis [9]. King et al. also showed that Lin28B can promote transformation of colonic epithelial cells and metastasis ability of colon cancer cells through let-7-dependent and -independent mechanisms [13]. Successively, several other studies also provided evidence for the involvement of Lin28B in colon cancer development and progression [14, 15]. Here, we have also confirmed the overexpression of Lin28B in colon cancer, and have showed that Lin28B expression is associated with lymph node metastasis of colon cancer, which is consistent with previous researches. However, we did not observed the correlation between Lin28B expression and poor prognosis of colon cancer patients based on the TCGA data. Additionally, we also observed the elevated expression of Lin28A in colon cancer, and found that Lin28A mRNA expression is associated with colon cancer metastasis. Consistently, our result of cell migration and invasion also demonstrated that both of Lin28A and Lin28B could promote the migration and invasion of colon cancer cells in vitro.

We have also compared the effects of Lin28A and Lin28B on the proliferation and apoptosis of colon cancer cells in vitro. We have showed that both Lin28A and Lin28B promotes the proliferation of colon cancer cells, however, the mechanism of Lin28A and Lin28B facilitating cell proliferation is different, Lin28A contributes to the transition of cell cycle from S phase to G2/M phase, whereas Lin28B facilitates the transition of cell cycle from both G1 phase to S phase and S phase to G2/M phase. The different mechanisms of Lin28A and Lin28B facilitating cell cycle transition may result from that both oncogenes regulate the expression of different cyclins and cell-cycle dependent kinases (CDKs). It has been reported that Lin28A promotes the expression of cyclin D1 and CDK2, both of them are necessary for the cell cycle transition from G1 to S phase [16], whereas Lin28B not only directly regulates the expression of cyclins and CDKs that are necessary for cell cycle transition from G1 to S phase [17], but also indirectly regulates the cell cycle process from S to G2/M phase by elevating the activity of insulin signaling pathway [18–20]. These results are helpful to explain our observations about the different role of Lin28A and Lin28B in promoting the cell cycle transition. Additionally, we previously demonstrated that over-expression of Lin28A promotes the apoptosis of colon cancer cells induced by 5-Fu [6]. In this study, we have confirmed the result and also showed that Lin28B has the same effect on cell apoptosis induced by 5-Fu, however, the mechanism underling both onco-proteins promoting cell apoptosis is different.

In conclusion, we have detected the expressional profiles and compared the functions of Lin28A and Lin28B in colon cancer tissues for the first time, and we have showed that both of them are co-expressed and have functional similarities, however, the molecular mechanisms underlying their similar functions may not be identical.

## MATERIALS AND METHODS

### Ethics statements

This study was approved by the Harbin Medical University Institutional Ethnic Committee. Written informed consent was signed by all patients enrolled for sampling and research. And all the methods in this study were carried out in accordance with the Helsinki Declaration of 1975.

### Tissues and patients

A total of 65 paraffin-embedded tissue samples and 10 normal colon tissues were obtained from patients with colon cancer, who received the operative treatment before other therapies in the affiliated tumor hospital of Harbin Medical University between May 2010 and June 2013. The diagnoses were confirmed by at least two pathologists. The integrated clinical pathological information of all patients is shown in Table 1. Additionally, 162 colon cancer patients with complete clinical information and whole genome wide gene expression data were downloaded from The Cancer Genome Atlas (TCGA). The information about the clinical pathological characters is shown in Table 2. Additionally, two colorectal adenocarcinoma data sets from TCGA (631 samples) and Genentech (72 samples) databases were employed to detect the mutation status of both oncogenes.

**Table 1:** The clinical characteristics of colon cancer patients from the affiliated tumor hospital of Harbin Medical University

Clinical parameters	Values
Mean age (range years)	59.5 (34-83)
Sex (Male/Female)	43/22
Differentiation (poor/moderate/well)	3/52/10
Lymph node metastasis	25/65 (38.5%)
Remote organ metastasis	0

**Table 2:** The clinical characteristics of colon cancer patients from TCGA

Clinical parameters	Values
Mean age (range years)	70.6 (36-90)
Sex (Male/Female)	81/81
Differentiation (poor/moderate/well)	Not available
Lymph node metastasis	63/162 (38.9%)
Remote organ metastasis	25/162 (15.4%)

### Immunohistochemistry

The expression of Lin28A and Lin28B at protein level in colon cancer tissues was detected by immunohistochemistry as previously described [6]. Briefly, 5-μm-thick colon cancer tissue sections were prepared. After deparaffinization, rehydration and blocking endogenous peroxidase, the antigen was retrieved by microwave treatment. The sections were blocked by using 5% bovine serum albumin and then incubated with primary antibodies against Lin28B (Abcam, Cambridge, MA, USA) or Lin28A (Abcam) at 4°C overnight. After incubation with secondary antibody labeled with streptavidin-biotin peroxidase for 1h at room temperature, DAB substrate (ZSGB Bio, Beijing, China) was applied for staining.

The result was evaluated by a pathologist who was blinded to the clinical information. The staining score was given by allying intensity with extent. Staining intensity was quantified as follows: negative (0), weak (1), moderate (2), or strong (3). Staining extent was scored according to the percentage of positive cells: none (0), <25% (1), 25-50% (2), 50-75% (3), or >75% (4). The final score was calculated as, the intensity score × the extent score.

### Cell culture

Colon cancer cell line HCT116 and SW620, lentivirus packaging cell line 293TN were cultured in Dulbecco's modified Eagle medium (DMEM; Hyclone, Logan, UT, USA) with 100 μg/ml streptomycin, 100 IU/ml penicillin and 10% fetal bovine serum (FBS; Invitrogen, Carlsbad, CA, USA) at 37°C in a humidified atmosphere containing 5% CO_2_.

### Lentivirus preparation and transduction

Lin28A and Lin28B over-expressed lentiviral vectors were constructed and the pseudo lentivirus was packaged as previous report [21]. Briefly, the ORF of Lin28A and Lin28B was cloned by using PCR and then inserted into pWPXL vector respectively, and then transfected 293TN cells together with packing vectors (pMD2.G and pSPAX2) to get pseudo lentiviral particles. The sequence of primers used for the construction of Lin28A ORF and Lin28B ORF is shown in Table 3. To generate stable lentivirus transduction cell lines, HCT116 cells were incubated with 10 MOI of GFP lentiviral particles, Lin28A or Lin28B over-expression lentiviral particles for at least 12h, then cultured for 72h before sorting GFP positive cells by flow cytometry.

**Table 3:** Primers used in this research

Primer name	Primer sequence (5′-3′)
let-7a RT	GTCGTATCCAGTGCAGGGTCCGAGGTATTCGCACTGGATACGACaactat
U6 RT	F: AAAATATGGAACGCTTCACGAATTTG
Let-7a Real-time PCR	F: TGAGGTAGTAGGTTG R: GTCGTATCCAGTGCAGGGTCCGAGGT
U6 Real-time PCR	F: CTCGCTTCGGCAGCACATATACT R: ACGCTTCACGAATTTGCGTGTC
Lin28A ORF	F: ggatccATGGGCTCCGTGTCCAACCAG R: acgcgtTTCTGTGCCTCCGGGAGCAG
Lin28B ORF	F: ggatccTTGATGGCCGAAGGCGGGGCTA R: acgcgtTTCCTTTTTTGAACTGAAGGCCCC

### Immunofluorescent staining

HCT116 and SW620 cells were seeded in a 6-well plate and cultured for 24h. The cells were fixed with 10% formalin and permeabilized with 0.1% Triton X-100. Rabbit anti-Lin28B polyclone antibodies (Abcam) were incubated with the cells at 37°C for 1h. After washing with PBS, the cells were incubated with the phycoerythrin (PE)-conjugated secondary antibody (Santa Cruz Biotechnology, Santa Cruz, CA, USA) at 37°C for 1 h. DAPI was used to highlight the nucleus.

### Western blotting

Total proteins were extracted from cells using RIPA buffer, and the nuclear and cytoplasmic proteins were respectively extracted using NE-PER^®^ Nuclear and Cytoplasmic Extraction Reagents (Thermo Fisher Scientific, Inc., Waltham, MA, USA). Proteins were separated by a 10% SDS-PAGE gel and then transferred to PVDF membranes (Bio-Rad, Hercules, CA, USA). The membranes were incubated primarily with anti-Lin28A (Abcam), anti-Lin28B(Abcam), anti-EGFP (Santa Cruz Biotechnology), anti-Lamin A(Abcam) or anti-Tubulin (Abcam) antibodies at 4°C overnight, and then incubated with the corresponding horseradish peroxidase-conjugated secondary antibodies (Santa Cruz Biotechnology, USA) at 37°C for 1h. Proteins were visualized by using an ECL enhanced chemiluminescence detection system (Thermo Scientific, Rockford, IL, USA). The images were captured by a Fujifilm Las-3000 imager (Fujifilm, Inc. Stamford, CT, USA).

### Quantitative real-time PCR

Total RNA was isolated using Trizol reagent (Invitrogen, Carlsbad, CA, USA). The RNA was reversed to cDNA using EasyScript^®^ Reverse Transcriptase (TransGen Biotech Co, Beijing, China). The level of let-7a was quantified by real-time PCR System (Bio-Rad) using a SYBR master mix (Roche, Indianapolis, IN, USA). The reaction mixture underwent 40 cycles consisting of denaturation for 10s at 95°C and annealing and prolongation for 30s each at 60°C. U6 was used as the endogenous controls for relative quantitation analyses. The sequence of specific primers is showed in Table 3.

### Cell growth assay

Luciferase-based ATP quantitation assay (CellTiter-Glo™, Promega, Madison, WI, USA) was used to detect the cell growth ability according to the manufacturer's instruction. First, the cells were seeded in 96-well plate at a density of 2×10^4^ cells per well. After cultured for 48h, 10 μl Cell Titer-Glo reagents was added to cell culture medium and incubated for 10min at room temperature. Then luminescence was determined by using a microplate reader.

### Colony formation assay

1 ml of 0.6% agarose gel with 1×DMEM complete medium was placed into 6 well-plates. Then 1×10^4^ cells suspended in 1ml of DMEM complete medium containing 0.3% agarose gel were inoculated on the surface of the solidified gel. These cells were continued to be cultured for 7 days at 37°C in a humidified atmosphere containing 5% CO_2_. The colony numbers were counted after the gels were stained with crystal violet.

### Invasion and migration assay

As previously described [22], cell invasion and migration were detected using transwell chambers with and without coated by matrigel (BD Bioscience, Sparks, MD, USA) according to the manufacturer's protocol. Briefly, 2×10^4^ cells suspended in 500μl serum-free medium were placed into the insert. Then 750μl complete medium was added to the 24-well chamber. Cells were incubated in 5% CO_2_ atmosphere at 37°C. After being cultured for 24 h, the cells were scraped on the upper surface of the insert, and the cells on the lower surface were fixed with formaldehyde and stained with hematoxylin. Under light microscopy, the average cell number was counted based on five randomly selected 200× fields.

### Cell apoptosis assay

The cell apoptosis was detected by using PE-Annexin V Apoptosis Detection Kit (BD Science, NJ, USA) as previously described [6].

### Statistical analysis

All these data were presented as the mean ± SD. The correlation between groups was detected by using Spearman correlation analysis. Comparisons between the groups were tested by Chi-square test, Student's t test or One-Way ANOVA analysis. P<0.05 was defined as statistically significance.
