# Correction: Comparison of the expression and function of Lin28A and Lin28B in colon cancer

**DOI:** 10.18632/oncotarget.27903

**Published:** 2021-02-16

**Authors:** Tianzhen Wang, Yan He, Yuanyuan Zhu, Mingwei Chen, Mingjiao Weng, Chao Yang, Yan Zhang, Ning Ning, Ran Zhao, Weiwei Yang, Yinji Jin, Jing Li, Riju James Rajkumar Ezakiel Redpath, Lei Zhang, Xiaoming Jin, Zhaohua Zhong, Fengmin Zhang, Yunwei Wei, Guomin Shen, Dong Wang, Ying Liu, Guangyu Wang, Xiaobo Li

**Affiliations:** ^1^ Department of Pathology, Harbin Medical University, Harbin, China; ^2^ Department of Gastrointestinal Medical Oncology, the Affiliated Tumor Hospital of Harbin Medical University, Harbin, China; ^3^ Department of Anatomy, Harbin Medical University, Harbin, China; ^4^ Department of Nutrition and Food Hygiene, Public Health College, Harbin Medical University, Harbin, China; ^5^ Department of Gastrointestinal Surgery, International Hospital of Pecking University, Beijing, China; ^6^ Department of Microbiology, Harbin Medical University, Harbin, China; ^7^ Department of General Surgery, the First Affiliated Hospital of Harbin Medical University, Harbin, China; ^8^ Department of Medical Genetics, Medical College, Henan University of Science and Technology, Luoyang, China; ^9^ College of Bioinformatics Science and Technology, Harbin Medical University, Harbin, China; ^10^ The Northern Medicine Translational Center, Heilongjiang Province Academy of Medical Science, Harbin, China; ^*^ These authors have contributed equally to this work

**This article has been corrected:** Due to errors during figure assembly, the images for Figure 1C and Figure 1F were accidentally switched. The corrected Figure 1, obtained using the original data, is shown below. The authors declare that these corrections do not change the results or conclusions of this paper.

Original article: Oncotarget. 2016; 7:79605–79616. 79605-79616. https://doi.org/10.18632/oncotarget.12869

**Figure 1:**
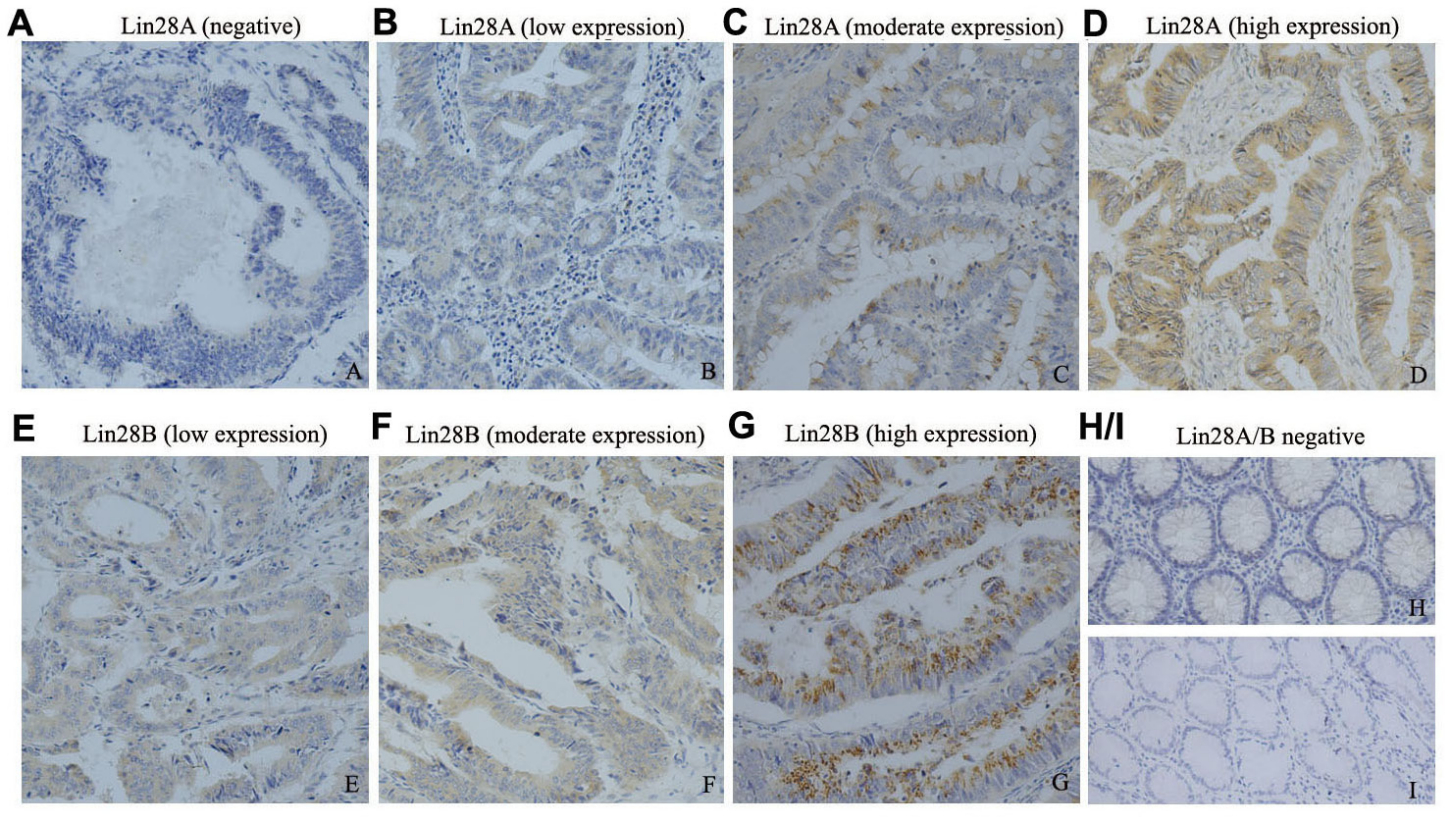
The expression pattern of Lin28A and Lin28B in colon cancer tissues detected by immunohistochemistry (200×). (**A**–**D**) illustrates the expression of Lin28A in colon cancer tissues variated from negative to high expression; whereas (**E**–**G**) illustrates the expression of Lin28B in colon cancer tissues variated from low to high expression (n=65). (**H**, **I**) illustrate the negative expression of Lin28A and Lin28B in normal colon tissues (n=10) respectively.

